# Impact of chronic illness caused by chikungunya fever on quality of life and functionality

**DOI:** 10.31744/einstein_journal/2024AO0562

**Published:** 2024-09-20

**Authors:** Jéssica Isabelle Santos Dutra, Marcelo Cardoso de Souza, Caio Alano Almeida Lins, Anna Cecília Queiroz de Medeiros

**Affiliations:** 1 Universidade Federal do Rio Grande do Norte Faculdade de Ciências da Saúde do Trairi Santa Cruz RN Brazil Faculdade de Ciências da Saúde do Trairi, Universidade Federal do Rio Grande do Norte, Santa Cruz, RN, Brazil.; 2 Universidade Federal do Rio Grande do Norte Department of Physiotherapy Natal RN Brazil Department of Physiotherapy, Universidade Federal do Rio Grande do Norte, Natal, RN, Brazil.

**Keywords:** Chikungunya fever, Epidemiology, Quality of life, Pain

## Abstract

**Objective:**

To evaluate the impact of chronic illness caused by chikungunya fever on the quality of life and functionality of affected individuals.

**Methods:**

A cross-sectional and comparative study was conducted in which two groups were investigated: a Chikungunya Group comprising 25 patients with chronic fever screened after 1 year of illness via a telephonic survey, and a Healthy Group comprising 25 healthy individuals matched for sex and age by face-to-face interview. The Stanford HAQ 20-Item Disability Scale (HAQ) and the Short Form Health Survey (SF-12) questionnaires were administered to both groups. Generalized Linear Models, Pearson χ^2^ tests, and odds ratios were used to evaluate the test results.

**Results:**

Significant differences in functional capacity and quality of life were observed between the Chikungunya and Healthy Groups. The chance of some impairment in functionality was also much higher in the Chikungunya Group in four of the HAQ categories, especially in the "walking" category (adjusted OR= 109.40). Further, the Chikungunya Group had a higher chance of presenting a below-average score in the mental component summary of the SF-12 (adjusted OR= 16.20) and of being positive in depression risk screening (adjusted OR= 34.57).

**Conclusion:**

Even one year after the acute phase, chikungunya fever can compromise the functionality and quality of life in affected individuals, with direct impairment in performing daily activities. Studies and therapeutic plans for chikungunya fever should consider the long-term impacts of this disease.

## In Brief

Chikungunya fever compromises the functionality and quality of life in the affected individuals, even one year after the acute phase of the disease. Chronically affected people experience direct impairment in performing daily activities, along with a risk of developing other morbidities.

## Highlights

◼ Even after a year, chikungunya fever-affected people experience damage to their physical and mental health.
◼ Positive screening for depression risk was 13.5 times more likely in chronically affected.
◼ Patients with chronic chikungunya fever had a 76 times higher risk of walking impairments.

## Graphical abstract

**Figure:**
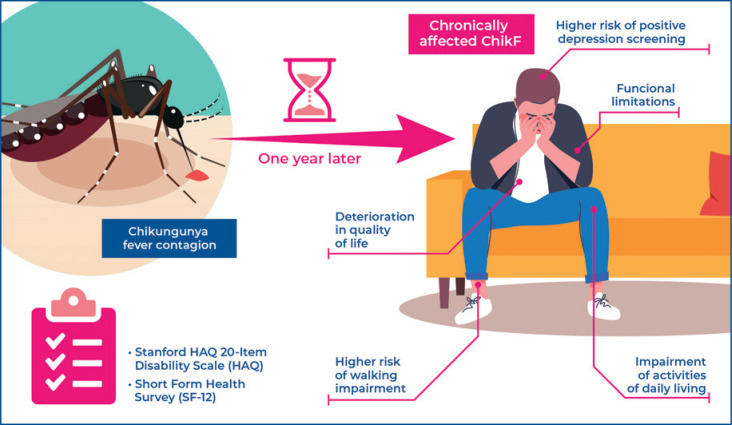

## INTRODUCTION

Chikungunya fever (ChikF) is an arbovirus transmitted by *Aedes aegypti* and *Aedes albopictus* mosquitoes, which are also dengue-transmitting vectors. It is characterized by sudden, intense, and severely debilitating polyarthralgia with high fever, intense myalgia, rash, headache, and other symptoms, which appear continuously or intermittently.^(1,2)^

Chikungunya fever is classified into three stages: acute, subacute, and chronic. In the acute phase, the patient presents classic viral symptomatology, as previously described. The subacute phase is characterized by worsening and persistent arthralgia along with other joint symptoms after 14 days of illness, while the chronic period is characterized by persistence of these symptoms for more than three months.^(2,3)^

This chronic stage is the main characteristic distinguishing ChikF from other arboviruses. Large joint involvement is the main sequela reported in this phase because of the development of several inflammatory symptoms such as pain, stiffness, and joint edema,^(4)^ which may affect functional capacity and quality of life, directly impacting the personal and professional lives of patients.^(5,6)^

Functionality is defined as the ability of a person to perform activities intrinsic to their daily life, enabling autonomy in their way of life.^(7)^ Some authors have reported that these limitations commonly affect patients with rheumatic problems.^(8,9)^ Therefore, evaluating these aspects is an important approach for understanding the limitations or deficiencies that ChikF may induce in these individuals.

Further, quality of life is a comprehensive concept that allows a broad assessment of the individual. It is considered an excellent indicator to evaluate various aspects related to health and well-being, as it covers functionality, aspects of physical and mental health, and spiritual, psychological, and social aspects, enabling the identification (for example) of the impact of a certain condition on their lives.^(10)^

The relationship between a decreased quality of life and the presence of pain is well-established in the literature;^(11,12)^ however, few studies have investigated the impact of chronic impairment by ChikF on quality of life and performance.^(13,14)^ These variables are considered important in clinical practice because of their direct influence on patients’ lives, particularly in chronic degenerative diseases.^(12)^

A ChikF epidemic occurred throughout Brazil in 2016, which constituted a major public health problem owing to the high incidence rates and number of recorded deaths.^(15)^ Therefore, the number of people who progressed to the chronic phase is believed to be high, and owing to a lack of consensus on the full repercussions of the chronic illness caused by ChikF, assessing its impact on the quality of life and functionality of affected individuals is necessary.

## OBJECTIVE

To evaluate the quality of life and functionality of individuals with chronic chikungunya infection, one year after the illness.

## METHODS

This was a cross-sectional comparative study on the impact of ChikF on the quality of life and functionality of chronically affected patients after 1 year of illness. Therefore, two groups were investigated: the Chikungunya Group (ChikG), comprising patients in the chronic phase of ChikF (at least one year of illness), and the Healthy Group, comprising people who did not have the disease and were matched for sex and age.

### Study sample

The ChikG sample was obtained from 2,796 suspected cases of ChikF in 2016, tracked, and reported by the Epidemiological Surveillance Center of the Zoonosis Control Center (CCZ) of Natal-RN, Brazil. For inclusion in the ChikG, the patient had to be >18 years of age, have confirmed ChikF (by laboratory or clinical-epidemiological criteria)^(2)^ and be in the chronic phase of the disease at the time of the interview. Individuals who presented incomplete records regarding case confirmation and telephone numbers and/or did not answer the telephone were excluded after three attempts, performed on different days and times.

**Figure 1:**
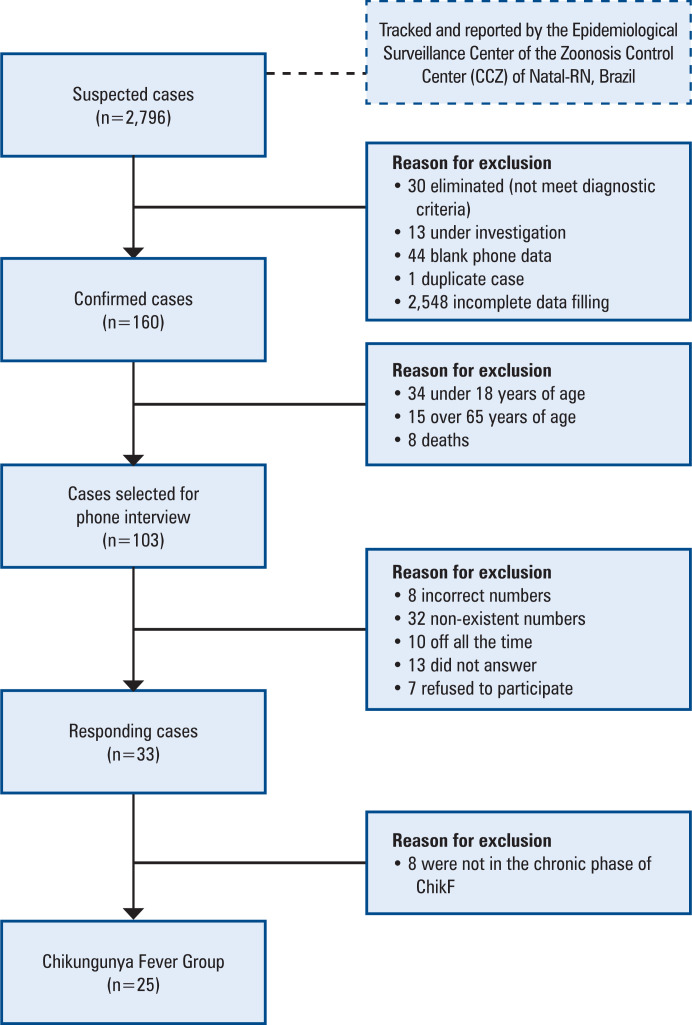
Flowchart showing the recruitment and selection of the Chikungunya Fever Group participants

After filtering according to the inclusion criteria, 103 patients contacted via telephone between January and February 2018 were selected to participate in the study. Of 33 patients who responded to the survey, 25 were in the chronic phase of the disease and comprised the Chik Group (Figure 1).

The Healthy Group comprised 25 adults recruited by convenience matching for sex and age who self-reported no suspicion of ChikF disease (previous or current) and no inflammatory joint disease. The exclusion criteria included non-acceptance of the study terms and residence in another federal state. The Healthy Group members were recruited from Natal and other municipalities in Rio Grande do Norte between June and July, 2018. To this end, 52 people were approached. Of these, 5 refused to participate in the study and 22 were eliminated because they did not match the ChikG in terms of sex or age.

### Data collection

Telephone data and socioeconomic characteristics of ChikG were collected from individual notification forms in the CCZ/Natal-RN records. Next, a telephone interview was conducted with the selected patients between January and February 2018, during which the Stanford HAQ 20-Item Disability Scale (HAQ) and SF-12v2 Short Form Health Survey (SF-12) questionnaires were administered (Brazilian Portuguese version). The telephone interviews took place at least one year after the disease notification record. Healthy Group data were collected between June and July 2018 through face-to-face interviews to characterize the participants’ socioeconomic classifications and to apply the HAQ and SF-12.

### Instruments

The HAQ is a self-administered questionnaire, which is widely used to assess functional capacity,^(16,17)^ enabling the assessment of an individual's overall function by analyzing the level of difficulty in performing daily activities. It comprises 20 questions divided into eight categories, and the calculated scores range from 0 (no difficulty) to 3 (cannot do).^(18)^ In the present study, the score of each category was transformed into a dichotomous variable: "uncompromised" (for those with the highest score equal to 0), and "compromised" (scores above 0). The HAQ has already been translated and validated for the Brazilian population and can also be administered through telephone interviews.^(19)^

The SF-12 is a generic quality-of-life questionnaire that is considered appropriate for different forms of application, including telephone administration, by a trained interviewer.^(20)^ It is a shorter version of the Medical Outcomes Study 36-Item Short-Form Health Survey (SF-36) and has been the instrument of choice in several population studies that assess quality of life because it is short and accurate.^(20–22)^ The SF-12 consists of 12 questions associated with the same eight health domains as those in SF-36: Functional Capacity, Social Aspects, Limitation by Physical Aspects, Emotional Aspects, Mental Health, Vitality, Body Pain, and General Health State.^(23)^

The scores for each domain as well as the physical (PCS) and mental (MCS) component summaries were calculated using the QualityMetric Health Outcomes Scoring Software 5.1. The program itself ranked each respondent as below-average and within or above average according to the component summary results. The program also assessed the participants for the risk of developing depression by tracking this risk from the outcome of the component summary of mental health (scores below 42). The software classified these items based on a comparison with the population data using an electronic algorithm.^(23)^

### Data analysis

The data were evaluated for their distribution using the Kolmogorov-Smirnov test, and an asymmetric distribution was found. Individuals with elementary and high school education were grouped for the tests.

Generalized linear models were used to evaluate the SF-12 and HAQ scores, adjusted for the variables of skin color and education. The χ^2^ test was performed to analyze the association between groups and the classification of the eight HAQ components; physical and mental component summaries and depression risk tracking (calculated from the SF-12). A binary logistic regression test was performed to calculate the gross and adjusted odds ratios (OR).

Data analysis was performed using the (SPSS) software for Windows, version 20.0. The significance level was set at p≤0.05.

### Compliance with ethical standards

The study was approved by the Human Research Ethics Committee of the Health Sciences College of Trairi, *Universidade Federal do Rio Grande do Norte,* CAAE: 75055317.7.0000.5568; #2.678.696. Informed consent was obtained from all participants included in the study.

## RESULTS

Females predominated (92%) in ChikG, and the median age was 38 years (p25-75 = 28.5-50.5). The characteristics of the study groups are presented in table 1.

**Table 1:** Sociodemographic data for the Chikungunya and Healthy Groups

Characteristics		ChikF Group	Healthy Group	p value
		n=25 (%)	n=25 (%)	
Gender				
	Female	23 (92)	23 (92)	
	Male	2 (8)	2 (8)	
Age range				
	18-30	7 (28)	6 (24)	
	31-50	12 (48)	13 (52)	
	51-64	6 (24)	6 (24)	
Skin color				
	White	5 (20)	20 (80)	<0.001*
	Non-white	14 (56)	5 (20)	
	Did not answer	6 (24)	0 (0)	
Education				
	Elementary	4 (16)	1 (4)	0.022*
	Middle school	7 (28)	5 (20)	
	High school	8 (32)	19 (76)	
	Did not answer or left blank	6 (24)	0 (0)	

* Statistical difference determined using the χ^2^ test. p<0.05.

ChikG presented lower scores in all eight dimensions that comprise the SF-12 in evaluating the quality of life when compared to those in the Healthy Group, with those who had the disease showing the lowest score in the Body Pain domain. No statistically significant differences were found between the groups in terms of vitality (Table 2).

**Table 2:** Median of SF-12 domain values and the HAQ score between the Chikungunya and Healthy Groups

Variables		Healthy Group		ChikF Group		Wald χ^2^	p value	95%CI
		Median	(p25-75)	Median	(p25-75)			
SF-12								
	Functional capacity†	57.06	49.19–57.06	41.32	33.45–49.19	21.41	<0.001*	7.29–18.03
	Social aspects†	56.90	48.01–56.90	48.01	30.22–48.01	6.83	0.009*	2.17–15.16
	Limitations by physical aspects†	57.46	51.11–57.46	49.00	38.42–53.23	7.48	0.006*	2.13–12.89
	Emotional aspects†	56.28	48.48–56.28	45.89	35.49–56.28	5.62	0.018*	1.09–11.45
	Mental health†	52.74	52.74–58.47	41.26	35.53–49.87	12.78	<0.001*	5.07–17.39
	Vitality†	58.90	49.07–58.90	49.07	39.23–58.90	2.48	0.115*	-1.39–12.84
	Body pain†	57.73	48.71–57.73	39.69	30.67–48.71	19.66	<0.001*	7.25–18.73
	General health state†	57.69	47.75–57.69	47.75	33.84–47.75	7.68	0.006*	2.12–12.37
HAQ Score‡		0	0-0	0.8	0.43–1.43	28.23	<0.001*	-1.20 – −0.55

* Statistical difference by the generalized linear model test. p<0.05;

† SF-12 Domains;

‡ Assessment of functional capacity. "Skin Color" and "Education" variables were used as covariates in the model.

When comparing the medians for the final HAQ score, a statistically significant difference was also observed between the groups, and the ChikG presented greater functional disability than that in the Healthy Group (p<0.001) (Table 2).

A significant association was found between ChikG and impairment in all HAQ categories (p<0.05). We also found an association between ChikG and "below average" physical and mental component summaries as well as positive screening for SF-12 depression (p<0.05) (Table 3).

**Table 3:** Frequency of values for the diagnosis of physical and mental component summaries and screening for the risk of depression based on the SF-12 and HAQ domains for the chikungunya and the Healthy Groups

Variables		Healthy Group	ChikF Group	OR_gross_ 95%CI	p value*	OR_adjusted_ 95%CI	p value**
		n=25 (%)	n=25 (%)				
SF-12							
Physical component†							
	Average or above	22 (88)	10 (40)	11	<0.001	6.57	0.041
	Below average	3 (12)	15 (60)	2.58–46.7		1.076–40.135	
Mental component†							
	Average or above	23 (92)	16 (64)	6.4	0.019	16.20	0.045
	Below average	2 (8)	9 (36)	1.23–34		1.058–248.273	
Screening for depression‡							
	Yes	1 (4)	9 (36)	13.5	0.005	34.57	0.024
	No	24 (96)	16 (64)	1.55–117.3		1.584–754.427	
HAQ							
Getting dressed§							
	Not compromised	23 (92)	15 (60)	7.66	0.008	NS	0.053
	Compromised	2 (8)	10 (40)	1.47–39.9			
Standing up§							
	Not compromised	22 (88)	9 (36)	13	<0.001	11.06	0.015
	Compromised	3 (12)	16 (64)	3–55		1.586–77.145	
Feeding yourself§							
	Not compromised	25 (100)	11 (44)	2.27	<0.001	NS	0.998
	Compromised	0 (0)	14 (56)	1.46–3.53			
Walking§							
	Not compromised	24 (96)	6 (24)	76	<0.001	109.40	0.001
	Compromised	1 (4)	19 (76)	8.41–686.5		7.311–1637.331	
Personal hygiene§							
	Not compromised	24 (96)	16 (64)	13.5	0.005	NS	0.065
	Compromised	1 (4)	9 (36)	1.55–117.1			
Reaching§							
	Not compromised	22 (88)	6 (24)	23.2	< 0.001	NS	0.998
	Compromised	3 (12)	19 (76)	5.1-105.7			
Gripping/holding§							
	Not compromised	21 (84)	9 (36)	9.3	0.001	19.22	0.012
	Compromised	4 (16)	16 (64)	2.43-35.8		1.909–193.503	
Other activities§							
	Not compromised	21 (84)	7 (28)	13.5	< 0.001	16.07	0.005
	Compromised	4 (16)	18 (72)	3.39-53.6		2.306–112.078	

* Statistical difference according to the χ^2^ test. p<0.05;

** Statistical difference by binary logistic regression (p<0.05);

† SF-12 Summary Components;

‡ SF-12 depression screening;

§ HAQ categories.

In the association analysis, the chance of presenting a below-average score in the physical and mental component summaries of the SF-12 in the ChikG was 11 and 6.4 times higher than that in the Healthy Group, respectively, and the chance of positive screening for depression risk was 13.5 times higher in this group. The chance of presenting some impairment for the HAQ categories was also higher in the ChikG, especially in the "walking" category (OR= 76) (Table 3).

After adjusted analysis, the ChikG score was significantly associated with the presence of impairment in four categories, lower quality of life scores, and positive screening for depression (p<0.05) (Table 3).

The chance of presenting a below-average score in the mental component summary of the SF-12 in the ChikG was even higher after adjustment, being 16.20 times higher than that in the Healthy Group; further, the chance of positive screening for the risk of depression was 34.57 times higher in this group. The chance of some impairment in functionality was also much higher in the ChikG after adjustment, especially in the "walking" category (adjusted OR= 109.40) (Table 3).

## DISCUSSION

The present study showed significant differences between ChikG and Healthy Groups in both functional capacity and quality of life after 1 year of illness in patients who developed the chronic form of the disease. The most compromised aspects were the HAQ Walking category and the SF-12 Body Pain domain.

Chronic pain is the most cited sequela of ChikF in multiple studies^(24–26)^ and is considered an important risk factor for disability, with a greater propensity to develop depressive symptoms in those who have pain affecting several regions.^(26)^ Further, some people may progress to pain with neuropathic features. This type of pain usually occurs because of injury or dysfunction of the nervous system and is described as a burning, stinging, or prickly sensation.^(27)^

Neuropathic pain has also been associated with lower quality of life indices and lower efficacy of traditional treatments,^(1,13)^ suggesting increased worsening of the clinical picture, especially regarding the onset of disability and other morbidities, making their treatment even more challenging.

Among the usual activities assessed using the disability questionnaire, patients with chronic ChikF were found to have a 76 times higher risk of walking impairment. Another study^(28)^ that evaluated gait, balance, and grip strength in older adults with post-chikungunya chronic arthralgia also found impairment in these aspects and suggested arthralgia as the cause of this impairment.

Considering these aspects, it is possible to identify that the treatment perspectives of these patients are also impaired, as in addition to walking being inherent to daily activities, it is also a type of physical activity that helps prevent limitations related to rheumatological symptoms^(6,29)^ and contributes to an improvement in the quality of life in people with depressive symptoms.^(30)^

High risks of impairment in other categories were also identified, such as reaching, personal hygiene and standing up, highlighting the magnitude of limitations experienced by these patients. The presence of compromised basic daily activities directly impacts the quality of life of these people, as they become increasingly dependent on others for care owing to functional decline.

In this context, ChikG also had a 13.5 times higher risk of being positively screened for depression; this result only highlights the fragility of these patients in relation to the possible development of mental illness and deterioration in their quality of life. Another study^(31)^ also identified a significant correlation between the presence of depressive symptoms and a lower quality of life, with more severe disability scores among females.

Thus, these results warn about the risk of developing other morbidities, with an impact on physical as well as mental health, which may lead to an even worse deterioration in the quality-of-life indices of this group, hindering their long-term recovery.

Regarding these aspects of mental health, another study^(32)^ found an association between functional disability and suicidal ideation, suicide attempts, and suicide deaths among older adults. This suggests that functional limitations in daily activities, whether associated with depression or not, may contribute to an additional risk of suicidal ideation, attempted suicide, and even death by suicide.

Thus, these findings are even more worrying considering that chronic illness caused by ChikF even affects younger people who have the same limitations that are not expected in this group, as these people are soon exposed to functional and mental health impairments, which can increase the number of people at risk for other aspects of suicide.

These characteristics also suggest a major economic impact on health systems.^(12,33)^ Active adults, owing to the sequelae of ChikF, may require expensive and specific care and services for many years because of the decline in functioning and quality of life, consequently impacting social security and the labor market.^(33,34)^ Some of these people may also develop disabilities, leading to reduced labor force participation and early retirement.

This study had some limitations that should be considered when interpreting its results. There is a possibility of selection bias, considering that the Healthy Group consisted of people with higher levels of education and white skin color than those in the ChikG; however, the groups were matched for sex and age, and the analyses were adjusted for these covariates. However, the sample represents only a small portion of the population; therefore, the results cannot be extrapolated to all residents.

Further, the study was based on self-reported signs and symptoms, with the possibility of respondents overestimating the reported symptoms. However, this bias may not have influenced the results because the questionnaires applied were valid and referred to a short period of time, a maximum of 4 weeks prior to the interview.

Finally, as this is an observational study, it is difficult to infer the aspects that cause disability and decrease the quality of life or to deepen the clinical and biological characteristics related to these aspects, as the study did not include the results and evaluations of specific examinations and population follow-up.

## CONCLUSION

Chikungunya fever compromises the functionality and quality of life of chronically affected individuals, with direct impairment of walking and personal hygiene, affecting their autonomy to perform daily activities. In addition, this group presents a higher risk of developing other morbidities, especially those related to mental health.

Furthermore, the impairments identified even after one year of illness indicate the need to carry out more long-term follow-up studies of people affected by chikungunya fever. Therefore, treatment trajectories must also consider the possible need for longer follow-up.

These results may help health professionals working with rehabilitation because the findings reported in this study enable a greater understanding of the difficulties faced by those affected by chikungunya fever, thus assisting in evaluating and treating these people.

Multidisciplinary integration within the research scope is also fundamental to develop follow-up studies on functionality and quality of life in chronic illnesses caused by chikungunya fever, seeking to identify risk factors for the development of comorbidities as well as to evaluate alternatives for physical rehabilitation and prevent new injuries.
